# Procalcitonin-guided diagnosis and antibiotic stewardship revisited

**DOI:** 10.1186/s12916-017-0795-7

**Published:** 2017-01-24

**Authors:** Ramon Sager, Alexander Kutz, Beat Mueller, Philipp Schuetz

**Affiliations:** 1 University Department of Medicine, Kantonsspital Aarau, Tellstrasse, CH-5001 Aarau, Switzerland; 2 Faculty of Medicine, University of Basel, Basel, Switzerland

**Keywords:** Procalcitonin, Pneumonia, Respiratory tract infection, Sepsis, Antibiotic stewardship

## Abstract

Several controlled clinical studies have evaluated the potential of the infection biomarker procalcitonin (PCT) to improve the diagnostic work-up of patients with bacterial infections and its influence on decisions regarding antibiotic therapy. Most research has focused on lower respiratory tract infections and critically ill sepsis patients. A clinical utility for PCT has also been found for patients with urinary tract infections, postoperative infections, meningitis, and patients with acute heart failure with possible superinfection (i.e., pneumonia). In these indications, PCT levels measured on hospital admission were found to substantially reduce the initiation of antibiotic treatment in low-risk situations (i.e., bronchitis, chronic obstructive pulmonary disease exacerbation). For more severe infections (i.e., pneumonia, sepsis), antibiotic stewardship by monitoring of PCT kinetics resulted in shorter antibiotic treatment durations with early cessation of therapy. Importantly, these strategies appear to be safe without increasing the risk for mortality, recurrent infections, or treatment failures. PCT kinetics also proved to have prognostic value correlating with disease severity (i.e., pancreatitis, abdominal infection) and resolution of illness (i.e., sepsis). Although promising findings have been published in these different types of infections, there are a number of limitations regarding PCT, including suboptimal sensitivity and/or specificity, which makes a careful interpretation of PCT in the clinical context mandatory. This narrative review aims to update clinicians on the strengths and limitations of PCT for patient management, focusing on research conducted within the last 4 years.

## Background

There are important limitations to consider when using conventional diagnostic markers, such as blood cultures and inflammatory markers (i.e., C-reactive protein [CRP]), for patients with a clinical suspicion of infection, particularly in regard to suboptimal sensitivity and specificity [1]. These limitations often leave physicians with ambiguity regarding the necessity of the use of antibiotic treatment. As a consequence, unnecessary and prolonged exposure to antimicrobial agents adversely affect patient outcomes (e.g., risk for *Clostridium difficile* infection), while inappropriate antibiotic therapy increases antibiotic resistance in patients, resulting in a public health threat. A growing body of evidence supports the use of the infection marker procalcitonin (PCT) to improve the diagnosis of bacterial infections and to indicate resolution of infection, thereby helping to monitor patients and guide antibiotic therapy.

**Fig. 1:**
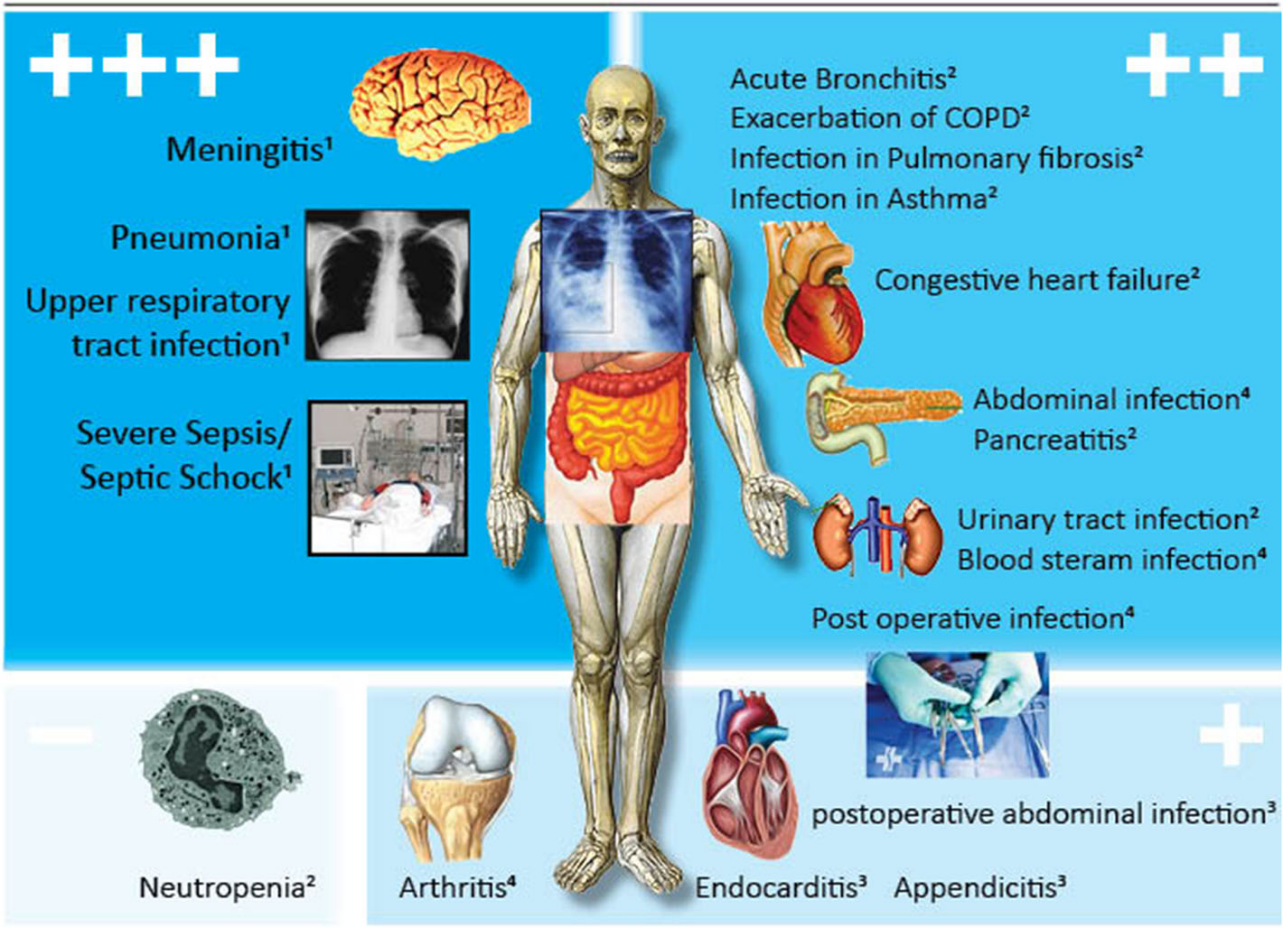
Summary of evidence regarding procalcitonin (PCT) for diagnosis and antibiotic stewardship in organ-related infections. While for some infections, intervention studies have investigated benefit and harm of using PCT for diagnosis and antibiotic stewardship (*left side*), for other infections only results from diagnostic (observation) studies are available (*right side*). +: moderate evidence in favor of PCT; ++: good evidence in favor of PCT; +++: strong evidence in favor of PCT; – no evidence in favor of PCT

Based on a review published in 2011 [2], this current narrative review aims to update clinicians on (promising) new indications for PCT in patient management by focusing on research studies published from 2012 to the end of 2016. The evidence from observational and interventional research is ordered by different types of infections and study designs. Proposed PCT cut-offs and the main conclusions of relevant studies are summarized in Table 1 and graphically displayed in Fig. 1.

**Table 1:** Overview of studies investigating the use of procalcitonin in different types and sites of infections

	Type of infection	New studies since 2010?	Study design	PCT cut-off (μg/L)	Benefit of PCT use?	Main conclusions	Selected references since 2012
Pulmonary	AECOPD	yes	RCT (N = 120), meta-analysis	<0.25	++	PCT reduces initiation of antibiotic treatment in the ED without adverse outcomes	[7, 12]
	Asthma	yes	RCT (N = 216)	<0.1–0.25	++	PCT reduces initiation of antibiotic treatment in the ED without adverse outcomes	[89]
	Bronchitis	yes (Registry)	RCT, real-life (Registry)	<0.1–0.25	++	PCT reduces initiation of antibiotic treatment in the ED without adverse outcomes	[42]
	Community-acquired pneumonia	yes	RCT, meta-analysis (N = 4467) real-life (Registry)	<0.1–0.25; 80–90% decrease	+++	PCT shortens length of antibiotic therapy in the ED and hospital ward without adverse outcomes	[7]
	Pulmonary fibrosis	yes	RCT (N = 78)	<0.25	++	PCT reduces initiation of antibiotic treatment in the ED without adverse outcomes	[15]
	Upper respiratory tract infections	no	RCT (N = 458, 702)	<0.1–0.25	+++	PCT reduces initiation of antibiotic treatment in primary care without adverse outcomes (non-inferiority)	[90, 91]
Heart	Congestive heart failure	yes	Observational, RCT (secondary analysis, N = 110)	<0.21–0.25	++	PCT helps in identification of bacterial superinfection in acute heart failure, may be helpful in guiding antibiotic treatment	[38, 43]
	Endocarditis	no	Observational, meta-analysis	<0.5	+	PCT is a predictor of poor outcome, diagnostic value similar to CRP	[67, 68]
Abdominal	Abdominal infections with peritonitis	yes	Observational	<0.5; 80% decrease	++	PCT-guided therapy was associated with lower antibiotic exposure with no difference in mortality	[66]
	Appendicitis	yes	Observational, meta-analysis	NR	+	PCT is a marker of complicated appendicitis, low value for diagnosing appendicitis	[92]
	Pancreatitis	yes	RCT (N = 71)	<0.5	++	PCT reduces antibiotic exposure compared to prophylactic antibiotic treatment without adverse outcomes	[65]
	Urinary tract infections	yes	RCT (N = 125)	<0.25	++	PCT reduces antibiotic exposure without adverse effects	[47]
Blood	Blood stream infection	yes	Observational	<0.25–1.47	++	PCT levels correlate with risk for positive blood cultures	[19, 27]
	Neutropenia	yes	RCT (N = 62)	NR	–	PCT is not useful to manage antibiotic therapy, but PCT was a marker of bacteremia	[93]
	Severe sepsis/shock	yes	RCT (N = 1575)	<0.5; 80% decrease	+++	PCT reduces antibiotic exposure and 3 month mortality in the ICU	[30]
Postoperative	Postoperative abdominal infection	yes	Observational, meta-analysis	NR	+	Low PCT post-surgical ensure safe discharge, PCT is similar to CRP	[58, 59]
	Postoperative infections	yes	RCTs, Observational	<0.5–2.0	++	Low PCT suggests absence of perioperative infection and enables early discharge	
Other	Arthritis	yes	Observational	<0.5	+	PCT identifies infection in patients with rheumatoid arthritis	[94]
	Erysipelas	yes	Observational	<0.1	+	PCT differentiates erysipelas from DVT	[81]
	Meningitis	no	RCT, meta-analysis (N = 2058)	<0.5	+++	PCT reduces AB treatment during viral outbreak; serum PCT with CSF lactate reliably identifies bacterial meningitis	[72, 74]

*Abbreviations*: *AB* antibiotic, *AECOPD* acute exacerbation of chronic obstructive pulmonary disease, *CSF* cerebral spinal fluid, *CRP* C-reactive protein, *ED* emergency department; *ICU* intensive care unit; *NR* not reported, *PCT* procalcitonin, *RCT* randomized controlled trial. The level of evidence in favor or against PCT for each infection was rated by two of the coauthors (PS, RSA) independently and disagreements were resolved by consensus. +: moderate evidence in favor of PCT; ++: good evidence in favor of PCT; +++: strong evidence in favor of PCT; –: no evidence in favor of PCT

### Infections of the respiratory tract

Infections of the respiratory tract, including bronchitis, community-acquired pneumonia (CAP), and acute exacerbated chronic obstructive pulmonary disease (AECOPD), are the most important drivers for antibiotic (over-) treatment and thereby contribute to the increasing rate of antibiotic multi-resistance. The diagnosis of respiratory infections relies mainly on clinical criteria (e.g., cough, dyspnea, fever) and chest radiography to document infiltration of the lung [3]. Identification of the pathogen by culture or polymerase chain reaction (PCR) methods has low sensitivity, and thus may not rule out bacterial infection [3]. For some types of infections, such as *Legionella*, clinical scores can help to assess the risk of infection [4, 5]. Several observational studies as well as interventional trials have established the usefulness of PCT in patients with respiratory infections.

**Fig. 2:**
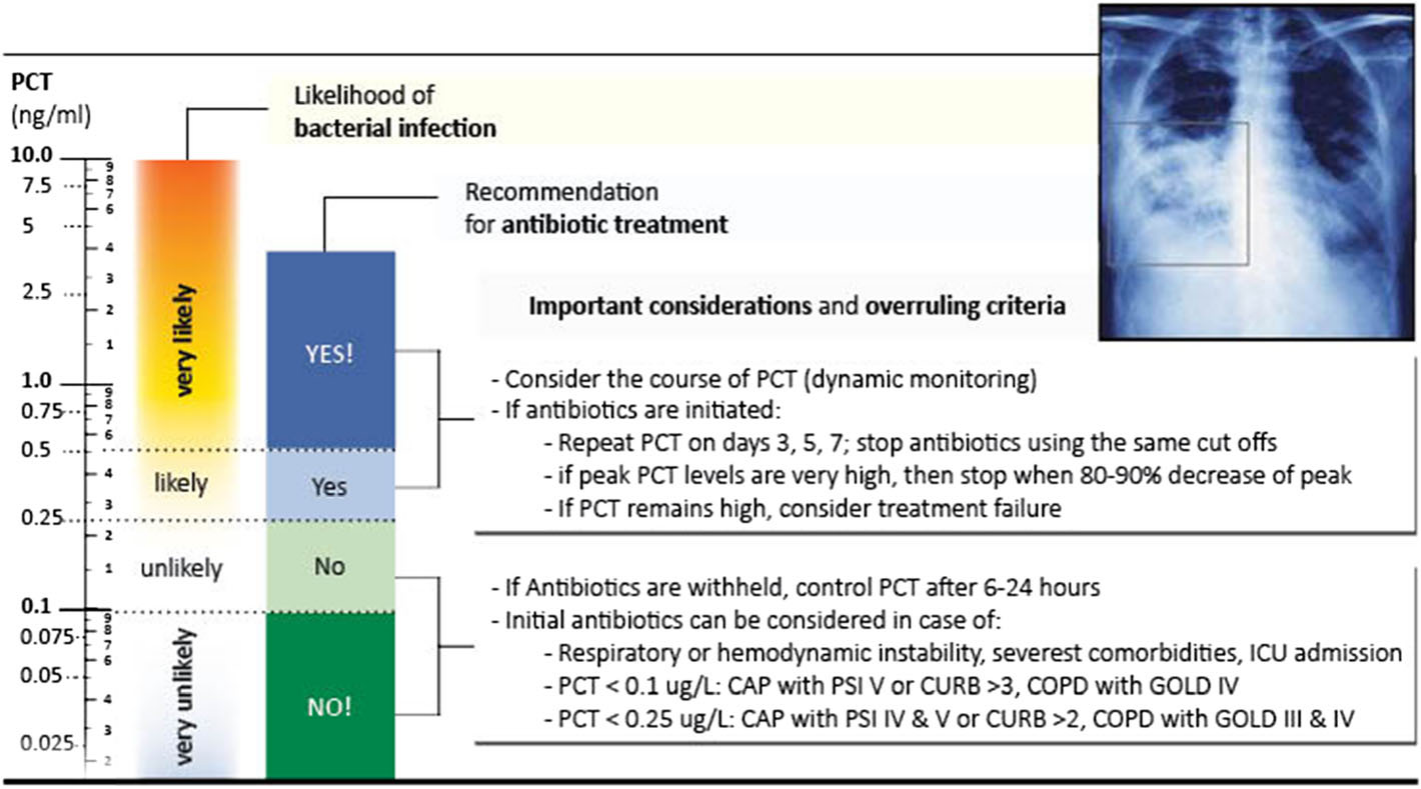
Procalcitonin (PCT) algorithm in patients with respiratory tract infections in the emergency department. The clinical algorithm for antibiotic stewardship in patients with respiratory tract infections in the emergency department encourages (>0.5 ng/ml or >0.25 ng/ml) or discourages (<0.1 ng/ml or <0.25 ng/ml) initiation or continuation of antibiotic therapy more or less based on specific PCT cut-off ranges. Abbreviations: *AB* antibiotic, *LRTI* lower respiratory tract infection, *ICU* intensive care unit, *PSI* pneumonia severity score

**Fig. 3:**
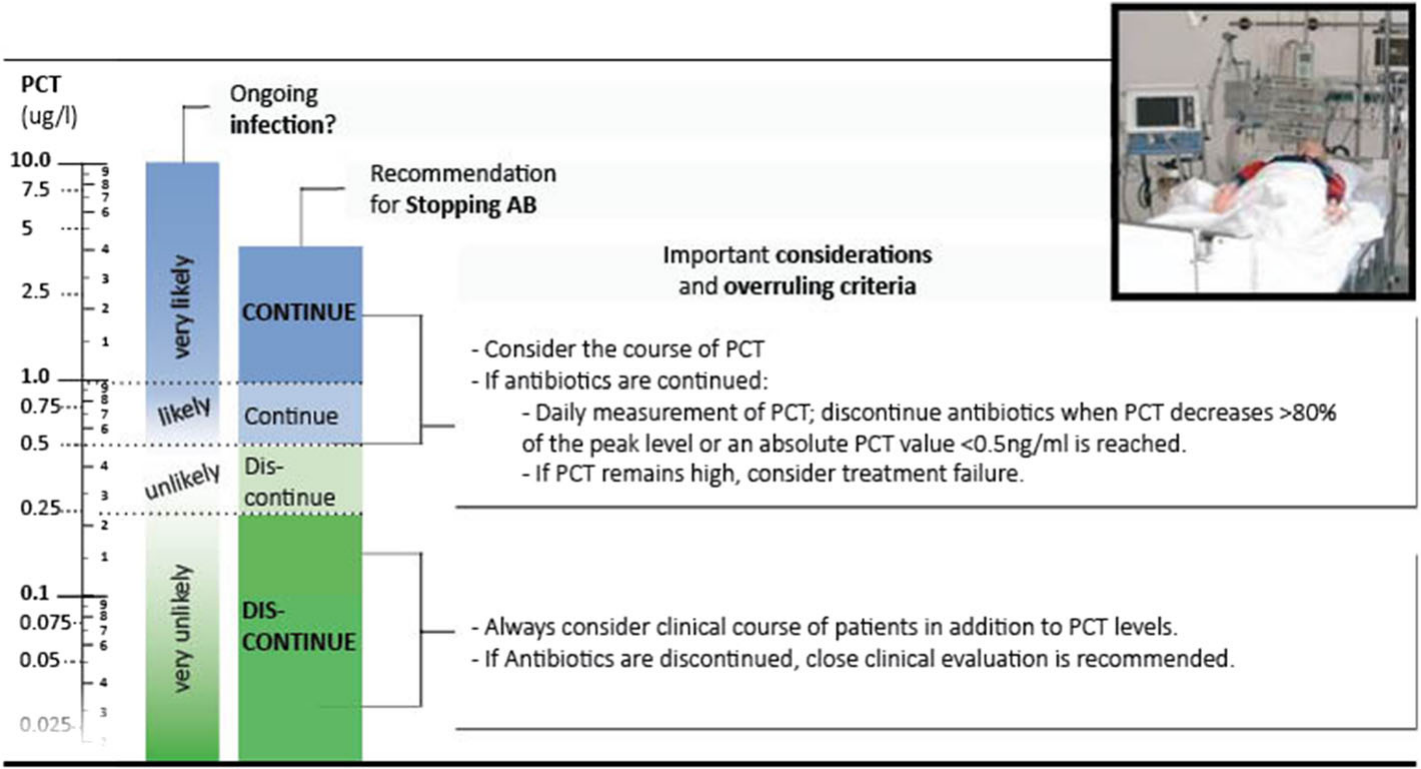
Procalcitonin (PCT) algorithm in patients with sepsis in the intensive care unit (ICU). In critically ill patients in the ICU, cut-offs are higher and initial empiric antibiotic therapy should be encouraged in all patients with suspicion of sepsis. PCT cut-offs are helpful in the subsequent days after admission to shorten the course of antibiotic therapy in patients with clinical improvement

A 2012 Cochrane meta-analysis based on individual patient data from 14 randomized controlled trials (RCTs) focused on effects of PCT in patients with respiratory infections [6, 7]. The studies had somewhat similar PCT protocols with recommendations for or against initiation or continuation of antibiotic therapy based on initial PCT levels, PCT kinetics over time (i.e., 80% PCT decrease from peak), or both, in combination with the clinical presentation and resolution of illness (Figs. 2 and 3) [8]. The cut-offs were adapted to the clinical setting and the acuity of presentation, with cut-offs of 0.25 (0.1) ng/ml in respiratory infections and 0.5 ng/ml in sepsis [9]. The meta-analysis found a strong reduction in the initial use of antibiotics, by around 60–70%, for low-severity respiratory infection (i.e., bronchitis, upper respiratory infections, AECOPD) [6, 9, 10]. In cases of higher-severity respiratory infection (i.e., pneumonia), monitoring of PCT resulted in earlier cessation of antibiotic treatment with a relative reduction in the duration of antibiotic treatment of around 40% for pneumonia and around 25% in the critical care of patients with sepsis caused by lung infections. Based on these studies, recent respiratory infection guidelines state that “… biomarkers can guide treatment duration by the application of predefined stopping rules for antibiotics. It has been shown that such rules work even in most severe cases, including pneumonia with septic shock …” [11], thereby emphasizing the concept of using a biomarker to guide the duration of antibiotic treatment.

An Italian RCT of patients with AECOPD published in 2016 found that a point-of-care PCT measurement was helpful in directing antibiotic therapy on admission [12]. In general, point-of-care technology has the advantage of high availability and short time to results for clinicians [13]. Patients randomized to the PCT group had a shorter antibiotic treatment course of 3.5 days compared to 8.5 for patients in the control group. The shorter duration in the PCT group did not negatively affect the resolution of illness or mortality.

Little research has so far focused on PCT use in patients with asthma and cystic fibrosis. A 2014 RCT in China of 216 patients with asthma also found PCT to be helpful in guiding antibiotic therapy, resulting in a reduction of prescribed antibiotics (48.9% versus 87.8%) and antibiotic exposure (relative risk 0.56, 95% confidence interval [CI] 0.44–0.70) [14]. There were no significant differences between the two groups in clinical recovery; length of hospital stay; or clinical, laboratory, and spirometry outcomes. For patients with idiopathic pulmonary fibrosis (IPF), a small RCT in China of 78 patients found a shorter treatment length (8.5 ± 6.6 days versus 14.2 ± 5.2 days) and fewer antibiotic prescriptions (26 versus 35 patients) in the PCT-guided group versus standard care. These small RCTs suggest that a PCT-guided protocol is superior to standard care in patients with suspected infection and underlying pulmonary disease (asthma, COPD, IPF) and reduces antibiotic consumption [15].

Novel studies have looked at the effect of PCT testing in “real life” in patients with respiratory infections. The ProREAL study included 1759 patients from Switzerland, France, and the USA and validated previous results from well-controlled RCTs [16, 17]. Importantly, compliance in trials is often higher compared to compliance in real life and post-study registries are thus important to understand how physicians use new diagnostic tools in clinical practice. Algorithm compliance in the ProREAL study overall was 68.2%, with differences between diagnoses, outpatients and inpatients, algorithm-experienced and algorithm-naive centers, and countries. Antibiotic therapy duration was significantly shorter if the PCT algorithm was followed compared with when it was overruled (5.9 versus 7.4 days; difference −1.51 days, 95% CI −2.04 to −0.98; *p* < 0.001). Another large propensity score-matched analysis investigated the impact of one to two PCT determinations on day 1 in the intensive care unit (ICU) on healthcare utilization and cost; this research database comprised 33,569 PCT-managed patients and 98,543 controls [18]. PCT utilization was associated with significantly decreased total and ICU length of stay, lower hospital costs, and lower total antibiotic exposure. Thus, real-life data showed similar effects compared to RCT data, emphasizing the importance of continuous education to increase knowledge and confidence in PCT protocols.

### Blood stream infection

Currently no gold standard exists for detecting blood stream infection (BSI). Yet, this is a key factor in targeted therapy and may improve survival. Blood culture is the most reliable diagnostic modality and samples for culturing are collected routinely in patients in the emergency department. Blood cultures give important information about the type of microorganism and its susceptibility to antibiotic treatment. However, only a small proportion of cultures give positive results and 40–90% remain negative [17, 19]. A large retrospective study from China found that only 440 of 2829 blood cultures (15.5%) were positive [20]. Furthermore, time to result is long, which precludes important initial treatment decision-making.

Multiple clinical scores and biomarkers have been investigated for their ability to predict blood culture positivity. A meta-analysis from 2013, including 3244 critically ill patients classified using the former definition of either sepsis or systemic inflammatory response syndrome (SIRS) of non-infectious origin, pooled the diagnostic power of PCT. Studies between 1996 and 2011 were included and showed that PCT had a high discriminatory ability (area under the curve [AUC] of 0.85), with pooled sensitivity and specificity of 0.77 and 0.79, respectively [21]. Similar results were reported in a large retrospective study from Korea in 2012 that included 3343 patients, showing an AUC of 0.76 for PCT in discriminating bacteremia from non-bacteremia [22].

In addition to biomarkers, clinical signs can also help in the assessment of the likelihood of culture positivity. One observational cohort study from 2015 that included 1083 patients who had blood culture drawn in the emergency department investigated several clinical scores and blood biomarkers, including the ability of PCT to predict blood culture sampling [19]. In blood culture-positive cases (N = 104, 9.6%) the Shapiro score and initial PCT concentration performed best, with AUCs of 0.729 and 0.803, respectively. Combining the Shapiro score and PCT significantly increased the AUC to 0.827. By limiting blood culture sampling only to patients with either a Shapiro score ≥4 or PCT >0.1 ng/ml would have reduced negative sampling by 20.2% while still identifying 100% of pathogens. A large retrospective study from 2016 in China including 2952 cases found an AUC of 0.713 for PCT, with an optimal cut-off value of 1.46 ng/ml for distinguishing negative from positive blood cultures (sensitivity 70%, specificity 64.5%) [20]. In contrast, an Austrian study published in 2014 that included 898 patients with 666 confirmed positive blood cultures (74.2%) found that PCT performed only moderately, with an AUC of 0.675. Even at a cut-off level of 0.1 ng/ml, PCT failed to correctly identify 46 positive blood cultures (7%) while reducing negative blood culture sampling by 20%. The researchers concluded that the false negative ratio was too high to implement blood culture sampling by PCT only [23]. These controversial results point out the need for further studies on this topic.

Interestingly, studies have also investigated PCT to assess whether blood cultures that test positive have been contaminated. Several studies found that PCT showed good discrimination between BSI and contamination with an AUC of 0.86 [22, 24]. Additionally, in a PCR diagnostic (SeptiFast) test, a PCT value of <0.37 ng/ml had a 99% negative predictive value for this assay [25]. Studies have also investigated how well PCT correlates with types of pathogens. Different PCT cut-off levels suggest different bacterial species, with higher concentrations for Gram-negative Bacteriaceae (AUC 0.81 at cut-off 6.47 ng/ml) [20, 26]. Furthermore, PCT has been shown to be helpful in differentiating infectious disease from autoimmune disease. In SIRS patients with negative blood cultures, PCT can differentiate septic from non-septic causes (AUC 0.892). A PCT cut-off value of 1.47–2 ng/ml shows the best performance (sensitivity 92%, specificity 83%) [27, 28].

### Sepsis, severe sepsis, and septic shock

Sepsis, as defined by SIRS criteria and organ dysfunction, and septic shock are common diseases in the ICU and require fast and appropriate antimicrobial therapy. Multiple studies have investigated whether a PCT-guided algorithm can optimize the therapeutic approach in these patients, mainly by monitoring PCT kinetics and stopping antibiotics once PCT has dropped to levels <0.5 ng/ml or by at least 80–90% of the peak in combination with clinical improvement. A 2013 meta-analysis including 1075 patients with sepsis or septic shock found overall reduced antibiotic treatment courses (6 days versus 8 days) when PCT was used to guide therapy compared to routine care. There was no increase in 28-day or in-hospital mortality or in length of stay in the ICU or the hospital. The authors do stress that there was heterogeneity in PCT protocols across trials with regard to different cut-off values or different algorithms for medical or surgical patients [29]. A large RCT published in 2016 in the Netherlands evaluated the use of PCT to de-escalate and stop antibiotics in 1575 critically ill patients who had received antibiotics <24 h before inclusion in the study for an assumed or proven infection [30]. The study found that the PCT-guided protocol shortened length of antibiotic treatment (5 days versus 7 days in the first 28 days of admission) and lowered 28-day mortality from 25 to 19.6% [30]. Although the cause of the reduced mortality remained unclear, a lower risk of antibiotic-associated side effects and improved therapeutic monitoring could have been factors [31]. Very similar results in a recently published RCT from Germany also found a lower antibiotic consumption using PCT, but no benefit in mortality [32].

Interestingly, a multicentre trial including 11 Australian ICUs and almost 400 patients found only a modest effect of PCT testing in regard to antibiotic reductions (median number of antibiotic treatment days 9 versus 11) [33]. The authors used a 0.1 ng/ml cut-off to stop antibiotic treatment, which may explain the differences to other sepsis trials that used a 0.5 ng/ml cut-off.

In addition, similar to other sepsis markers, PCT has been found to predict the severity of illness. A meta-analysis from 2015 showed a significant difference in PCT on day 1 and 3 between survivors and non-survivors. But cut-off values varied between studies and it was not possible to pool data to a common cut-off value. There have been several trials investigating prognostic scores to determine the severity of SIRS, such as the Predisposition, Insult, Response, Organ dysfunction (PIRO) model and the Mortality in Emergency Department Sepsis (MEDS) score [34, 35]. An important consideration in septic patients is that renal impairment and a reduced glomerular filtration rate may lower PCT clearance and levels thus may be higher than expected [36, 37].

### Congestive heart failure

Patients with congestive heart failure (CHF) often present to the ED with dyspnea. It can be challenging to differentiate acute heart failure from a lung infection. PCT may be helpful in this indication for ruling in or ruling out an infection [38–41]. PCT has thus been proposed as a promising marker for cardiologists [41]. A large study from China found that the severity of CHF was also a predictor for PCT levels in patients with infection complicated by heart failure [40]. A large observational study found a worse outcome in patients with a diagnosis of CHF and an elevated PCT concentration (>0.21 ng/mL) if they were not treated with antibiotics (*p* = 0.046). Patients with low PCT values (<0.05 ng/mL) had a better outcome if they did not receive antibiotic therapy (*p* = 0.049). Similar results were also found in a secondary analysis of a previous randomized trial and it was speculated that more appropriate treatment decisions due to PCT monitoring could explain this effect (diuretic therapy with liquid restriction in case of acute heart failure versus antibiotics and fluids in case of infection) [42, 43]. The role of PCT in the setting of decompensated CHF has potential and needs to be elucidated in future studies. Results from the IMPACT-EU trial may be of high relevance (https://clinicaltrials.gov/ct2/show/NCT02392689).

### Urinary tract infection

Several new publications have focused on the utility of PCT in urinary tract infection (UTI) with or without sepsis [44–46]. A threshold of 1.16 ng/ml was proposed by Julian-Jemenez et al. [44] as having the largest area under the receiver operating characteristic curve at 0.993 and therefore the most relevant in guiding medical decision-making [44]. The utility of PCT in this setting was also investigated in a Swiss RCT [47]. The study showed a reduction in antibiotic use of 30% compared to the standard treatment. The elaborated algorithm combined serum PCT concentration and quantitative pyuria measurements [47]. Patients were classified with uncomplicated or complicated UTI and as outpatients versus inpatients, resulting in different antibiotic administration, different length of treatment, or a monitored approach with measuring of PCT and degree of pyuria. There were no negative effects. The authors concluded that a PCT/pyuria-based approach is safe in terms of outcome and has the potential to reduce antibiotic consumption.

### Postsurgical infection

A physiological rise in PCT concentration is observed in postoperative patients due to surgical stress-triggered inflammation. The highest values are measured on the second postoperative day and usually show a sharp decline thereafter. Therefore, very high initial levels or non-dropping levels of PCT suggest postsurgical infection [48]. A chronological sampling was shown to be useful and superior to on-time sampling and predicted complications in the postoperative course [49]. Positive results for PCT were shown in studies of cardiopulmonary surgery, open aortic repair surgery, hip replacement, and liver transplantation [48–52]. Dong et al. found that PCT had a high discriminative power between septic and non-infective SIRS after cardiac surgery (cut-off value 0.47 ng/ml) [53]. PCT was also found to be useful to confirm surgical success after necrotizing fasciitis [54]. In complicated abdominal infection after surgery, high PCT correlates with mortality rates [55]. In addition, PCT is also beneficial in the setting of postoperative fever [56]. Several observational studies have questioned the reliability of PCT in postoperative patients with intra-abdominal infection. Although the negative predictive value was found to be high, which may help to ensure early discharge [57], the specificity was quite low. Also, a meta-analysis including 2692 patients after colorectal surgery showed no advantage of PCT compared to CRP regarding diagnostic accuracy and mortality prediction [58]. This was confirmed recently in a similar large prospective study including 501 patients [59]. Thus, further investigation is needed in the surgical setting, and also to compare the accuracy and cost-effectiveness of PCT with other infection markers such as CRP.

### Abdominal disease and abdominal infection

Several studies have investigated the use of PCT in patients with abdominal infection. In patients with liver cirrhosis, one study found that PCT could identify complications through bacterial infection [60, 61]. Similar results were found for acute pancreatitis, with PCT a good predictor of accompanied perforation or abscess [62–64]. In a small RCT of patients with acute pancreatitis, a PCT-guided antimicrobial approach was shown to be safe and effective compared to a control group that received prophylactic antibiotic treatment for 2 weeks [65]. Furthermore, in patients presenting with secondary peritonitis, PCT-guided therapy was shown to reduce antibiotic consumption by 50% [66]. There are currently no studies evaluating the role of PCT in infected diverticulosis and more studies are strongly needed in general to evaluate the use of PCT-guided treatment in abdominal infection.

### Endocarditis

Few studies have investigated the use of serum PCT in infectious endocarditis. Nevertheless, one large meta-analysis by Yu et al. found that PCT was not superior compared to CRP, and therefore not suitable in routine diagnostic [67]. However, Cornelissen et al. found PCT useful in the prediction of poor outcome (cut-off 0.5 ng/ml, sensitivity 73%, specificity 79%), with an odds ratio of 12.8 (95% CI 2.5–66.2) for finding *Staphylococcus aureus* in blood cultures [68].

### Febrile neutropenia

In patients with haematological malignancies with newly developed febrile neutropenia (FN), PCT is an accurate marker of infection as well as a predictor of severity [69, 70]. A 2015 meta-analysis provided the first large-scale clinical evidence on the validity of PCT in discriminating bacterial infection from other cause of fever in patients with FN [71]. In this study of 1960 febrile episodes, a positive likelihood ratio of 5.49 was found for PCT being a good marker for infection. Overall, PCT had a specificity of 0.88 but low sensitivity of 0.65, resulting in a suboptimal negative likelihood ratio of 0.4. Taken together, PCT might be specific, but not sensitive, in differentiating severe bacterial infection from other systemic inflammation or viral infection. The authors state that, with regards clinical implications, the use of PCT is valuable as a diagnostic aid to confirm infection. However, the negative likelihood ratio (0.4) is unacceptably high for guiding antimicrobial therapy and PCT is therefore not suitable for predicting treatment cessation in patients with FN. Moreover, a recent study showed a significant correlation between initial PCT concentration and ICU admission (AUC 0.93) and mortality (AUC 0.86) [70].

### Meningitis

Several older studies evaluated PCT-guided therapy in meningitis and determined that PCT-guided therapy reduces antimicrobial consumption during a viral outbreak [72]. Two recent meta-analyses confirmed PCT’s accuracy in differentiating viral from bacterial meningitis [73, 74]. The most recent meta-analysis from 2016 included 2058 subjects and showed a sensitivity of 0.95 (95% CI 0.89–0.97), a specificity of 0.97 (95% CI 0.89–0.99), a positive likelihood ratio of 31.7 (95% CI 8.0–124.8), and a negative likelihood ratio of 0.06 (95% CI 0.03–0.11). The diagnostic performance was even better when combined with cerebrospinal fluid lactate. Serum PCT was found to be more sensitive and specific than cerebrospinal fluid PCT [74]. Furthermore, PCT was useful for prognostication of poor outcome, follow up of treatment, and for differentiating from tuberculous meningitis [75].

### Septic arthritis

Several small studies have investigated the role of PCT in septic arthritis and bacterial osteomyelitis. In particular, in patients with rheumatologic disease, the differentiation between acute rheumatoid flare and infectious arthritis is of upmost importance. PCT was found to be a reliable marker at a cut-off of 0.4 ng/ml and is indicated especially if arthrocentesis cannot be performed [76]. The same is true in acute gouty arthritis, because PCT rises together with aseptic-gouty inflammation and can therefore be falsely positive [77, 78]. A PCT value beyond 0.5 ng/ml does not, however, rule out bacterial infection and appropriate treatment may be indicated [79]. Otherwise, PCT accuracy is highest when measured in fresh joint fluid [80]. We found no new studies evaluating PCT-guided antibiotic therapy in septic arthritis.

### Erysipelas

Few studies have investigated the use of PCT in local infections such as erysipelas. Early differentiation from similarly presenting deep vein thrombosis (DVT) based solely on clinical signs and symptoms is challenging. A 2015 study in Switzerland investigated the use of PCT to help physicians in discriminating between these infections. At a cut-off value of 0.1 ng/ml, PCT had a sensitivity of 0.57, a specificity of 0.82, and a positive predictive value of 0.86. Levels of PCT also correlated with the severity of erysipelas, with a stepwise increase according to SIRS criteria. Thus, PCT was found to be highly discriminant for differentiation between erysipelas and DVT but further research is warranted [81].

### Limitations

This narrative review has limitations. First, we did not do a systematic review for each type of infection but have selected studies based on a PubMed search and the authors’ expertise. Our conclusions may thus be too enthusiastic. Second, most of the studies did not blind patients and/or investigators and thus are subject to possible bias. Third, we focused on studies published between 2012 and mid-2016. Papers before or after this time frame may have been missed. We have also not discussed in detail other markers of infection such as CRP. However, there is a lack of well-done and large studies comparing the effect of both markers when used in the context of antibiotic stewardship [82, 83].

Importantly, PCT is far from being a perfect marker and levels must be evaluated in the context of a careful clinical and microbiological patient assessment. Because the kinetics of PCT are of particular diagnostic and prognostic importance, repeated measurements should be performed. This is particularly true for persistently sick patients and in situations in which antibiotics are withheld. The limitations of PCT include false-positive and false-negative results [84]. PCT levels can increase in the absence of a bacterial infection in patients with severe trauma or after surgery [84–86]. Here, PCT usually shows a rapid decline in follow-up measurements when the patient recovers. Also, patients with chronic renal failure can have a slower decrease in PCT levels. PCT levels can also be low in the early course or localised state of an infection, with later measurements showing an increase in levels. Again, repeated PCT measurements are advised in case of uncertainty. Another important consideration is the cost of measuring PCT. While some reviews found PCT to be cost-efficient in respiratory infections when antibiotics can be reduced by the measurement of this marker [87], this may not be true for other indications.

### Summary, future directions, and conclusions

This update of a previous narrative review found several interesting new clinical settings in which PCT-guided therapy could help to reduce antibiotic exposure by decreasing either initiation or duration of treatment. Particularly, controlled clinical studies have found that PCT improves the management of patients with lower respiratory tract infections and critically ill sepsis patients, as well as patients with UTIs, postoperative infections, meningitis, and acute heart failure with possible superinfection (i.e., pneumonia). Recording PCT levels on hospital admission was found to substantially reduce the initiation of antibiotic treatment in low-risk situations (i.e., bronchitis, AECOPD). For more severe infections (i.e., pneumonia, sepsis), antibiotic stewardship by monitoring PCT kinetics resulted in shorter antibiotic treatment duration by early cessation of therapy. These strategies appear to be safe without increasing the risk for mortality, recurrent infections, or treatment failure. PCT kinetics have also proved to have prognostic value, correlating with disease severity (i.e., pancreatitis, abdominal infection) and resolution of illness (i.e., sepsis). While there is strong evidence regarding antibiotic stewardship in respiratory infection and sepsis, PCT has not been as well studied for other types of infections. Thus, future research should focus on non-respiratory infections and investigate whether PCT improves antibiotic decisions in these patients. PCT should also be compared to other markers, such as CRP, in regard to diagnostic accuracy and cost-effectiveness.

Emerging bacterial resistance to antimicrobial agents calls for more effective efforts to reduce the unnecessary and prolonged use of antibiotics in self-limiting non-bacterial and resolving diseases [88]. Healthcare professionals share a common goal of achieving symptom relief from infection as fast as possible and often see antibiotics as the best means to achieve this goal. This “one size fits all” approach, however, does not answer the basic questions of who truly benefits from antibiotic therapy and what would be the optimal duration of treatment. There is a growing body of literature supporting the use of PCT as a persuasive, evidence-based approach to a more rational use of antibiotics.
